# Development of Multiplex Molecular Assays for Simultaneous Detection of Dengue Serotypes and Chikungunya Virus for Arbovirus Surveillance

**DOI:** 10.3390/cimb46030134

**Published:** 2024-03-06

**Authors:** Louis Robert W. Belem, Sylvester Agha Ibemgbo, Michel Kiréopori Gomgnimbou, Dileep Kumar Verma, Antoinette Kaboré, Ankit Kumar, Ibrahim Sangaré, Sujatha Sunil

**Affiliations:** 1 Vector Borne Diseases Group, International Centre for Genetic Engineering and Biotechnology, New Delhi 110067, India; louisro2007@yahoo.fr (L.R.W.B.); silver.ibm@gmail.com (S.A.I.); dileep21feb@gmail.com (D.K.V.); ankitkumar.bcas@gmail.com (A.K.); 2 Centre d’Excellence Africain en Innovations Biotechnologiques pour l’Elimination des Maladies à Transmission Vectorielle (CEA/ITECH-MTV), Université Nazi Boni, Bobo-Dioulasso 01 BP 1091, Burkina Faso; gomikir@yahoo.fr (M.K.G.); babaibrasangare@yahoo.fr (I.S.); 3 Ecole Doctorale Sciences Naturelles et Agronomiques (ED-SNA), Université Nazi Boni, Bobo-Dioulasso 01 BP 1091, Burkina Faso; 4 Laboratoire de Recherche, Centre MURAZ, Institut National de Santé Publique, Bobo-Dioulasso BP 10278, Burkina Faso; 5 Institut Supérieur des Sciences de la Santé (INSSA), Université Nazi Boni, Bobo-Dioulasso 01 BP 1091, Burkina Faso; 6 Laboratoire National de Référence, Institut National de Santé Publique, Ouagadougou BP 10278, Burkina Faso; awskabore@yahoo.fr; 7 Centre Hospitalier Universitaire Souro Sanou (CHUSS), Bobo-Dioulasso 01 BP 676, Burkina Faso

**Keywords:** multiplex, molecular, detection, dengue virus, chikungunya virus, arbovirus

## Abstract

The major arboviruses mainly belong to the *Bunyaviridae*, *Togaviridae*, and *Flaviviridae* families, among which the chikungunya virus and dengue virus have emerged as global public health problems. The main objective of this study was to develop specific, sensitive, and cost-effective molecular multiplex RT-PCR and RT-qPCR assays for the rapid and simultaneous detection of CHIKV and the four serotypes of DENV for arbovirus surveillance. Specific primers for all viruses were designed, and one-step multiplex RT-PCR (mRT-PCR) and RT-qPCR (mRT-qPCR) were developed using reference strains of the CHIKV and DENV serotypes. The specificity of the test for all the viruses was confirmed through sequencing. The standard curves showed a high correlation coefficient, R^2^ = 0.99, for DENV-2 and DENV-3; R^2^ = 0.98, for DENV-4; and CHIKV; R^2^ = 0.93, for DENV-1. The limits of detection were calculated to be 4.1 × 10^−1^ copies/reaction for DENV-1, DENV-3, and CHIKV and 4.1 × 10^1^ for DENV-2 and DENV-4. The specificity and sensitivity of the newly developed mRT-PCR and mRT-qPCR were validated using positive serum samples collected from India and Burkina Faso. The sensitivity of mRT-PCR and mRT-qPCR are 91%, and 100%, respectively. The specificity of both assays was 100%. mRT-PCR and mRT-qPCR assays are low-cost, and a combination of both will be a useful tool for arbovirus surveillance.

## 1. Introduction

The major arboviruses belong to the families *Bunyaviridae*, *Togaviridae*, and *Flaviviridae* [1]. Arboviruses are mainly transmitted by *Aedes aegypti* and *Aedes albopictus* [2,3,4]. Yellow fever virus (YFV), dengue virus (DENV), Zika virus (ZIKV), West Nile virus (WNV), and chikungunya virus (CHIKV) are arboviruses globally distributed [5,6]. High mutation rates among RNA arboviruses provide conditions for adaptive evolution to new mosquito species, which may facilitate arbovirus disease emergence [7]. DENV and CHIKV are arboviruses of the family *Flaviviridae* and *Togaviridae*, respectively. DENV is the most prevalent arbovirus, present in more than 100 countries in the world [8]. This virus is an enveloped single-stranded, positive-sense RNA virus, comprising four antigenically distinct serotypes, DENV-1 to DENV-4 [2,3]. CHIKV has now been identified in over 110 countries in Asia, Africa, Europe, and the Americas [9] and is also a positive-sense single-stranded RNA virus with a genome of approximately 11.8 kb [10]. Moreover, when the multiple serotypes of DENV circulate concurrently with CHIKV, there is a higher risk for more severe forms of the disease, such as dengue hemorrhagic fever (DHF) and dengue shock syndrome (DSS) [11].

Between 2014 and 2017, 28.4% of dengue cases were serologically confirmed in India [12]. In 2016, followed by 2017 and 2019, the maximum number of chikungunya laboratory-confirmed cases were reported in India. The highest confirmed cases were reported in Karnataka, Delhi, and Maharashtra [13]. In Burkina Faso, DENV is the most widespread arbovirus, with the highest prevalence, incidence, and significant morbidities and mortality. In 2016, more than 1061 cases of dengue were reported in Burkina Faso, with a case fatality rate of 1.2% [14]. In 2017, a prevalence of 28.54% of dengue fever was reported among pregnant women in Ouagadougou [15]. Another sero-epidemiological study of CHIKV was conducted in Ouagadougou, Burkina Faso, with blood samples collected in 2015, and a seroprevalence of 29.1% was reported [16]. These consecutive reports in different populations (general and pregnant populations) suggest a considerable dengue burden in Burkina Faso. The risk of arbovirus transmission in the country is real because of its tropical climate, which favors the multiplication of *Aedes*. Almost 80% of the Burkinabe population engages in farming and/or other agricultural practices. This may constitute a major factor in arbovirus transmission because, during the rainy season, farmers settle in the fields close to the forest and are in closer contact with vectors, which increases the risk of arbovirus transmission [17,18]. In the absence of a vaccine and specific antiviral treatment against DENV and CHIKV, the most effective means of disease control remain surveillance for early detection of cases in order to intervene with public health measures to contain the cases and thus control the infection.

The initial disease symptoms manifested by chikungunya and dengue are classical, such as high fever, headache, and nausea [19], and overlap with other acute febrile illnesses such as COVID-19, flu, and malaria. Moreover, chikungunya and dengue have similar symptoms, such as myalgia and arthralgia, making differential diagnosis clinically difficult, and currently, molecular diagnostic tools that are both specific and sensitive to confirm differential diagnosis of CHIKV and DENV serotypes are still lacking. Serological testing for these infections has well-documented limitations: antibodies to immunoglobulin M (IgM) may not be detectable early in the course of infection; a rise in immunoglobulin G (IgG) between acute and convalescent samples can only provide a retrospective diagnosis; and anti-flavivirus antibodies may cross-react with one another [20]. IgM and IgG ELISA tests are widely used for rapid serological diagnosis but have the limitation of the inability to identify the circulating viral serotypes [21]. Virus isolation and amplification in susceptible cell lines is the gold standard for the detection of serotypes, but it is not an appropriate clinical diagnostic assay for early infection since it is laborious and time-consuming [22]. For the prevention and detection of local transmission of DENV and CHIKV via both human and mosquito surveillance, developing a highly specific, sensitive, and less expensive detection method with rapid outcomes appears particularly important. Multiplexed PCR-based assays often fail because multiple primers presented in high concentrations interact with each other unless they are exquisitely designed. Non-specific interference of oligonucleotides (DNA and RNA) is also thought to limit further multiplexed PCR [23]. Many molecular methods have been developed to improve the simultaneous detection of several arboviruses around the world [11,24,25,26,27]. The present study was conducted to develop a one-step multiplex reverse transcription polymerase chain reaction (mRT-PCR) and a real-time polymerase chain reaction (mRT-qPCR) for the rapid and simultaneous detection and serotyping of DENV and CHIKV to be used in arbovirus surveillance in Burkina Faso.

## 2. Materials and Methods

### 2.1. Viruses

Isolates of DENV and CHIKV were used for the development of multiplex PCRs and their analytical performance determination. Reference strains of DENV 1–4 (1 isolate per serotype) were obtained from ATCC (bei RESOURCES, Manassas, Virginia, USA)(VR-1856™ DENV-1; VR-1584™ DENV-2; VR-1256_FD™ DENV-3; and VR-1490™ DENV-4) and CHIKV was isolated from a PCR-positive human sera sample [28]. The viruses were propagated in C6/36 *Aedes albopictus* cells prior to viral RNA extraction. Briefly, 20 µL of the isolated sera sample was mixed with DMEM with 2% FBS, filtered, and inoculated in confluent 24-well plates of C6/36 cells, followed by incubation for 6 days. Approximately 90–100% confluent 6-well plates of Vero cells were used for CHIKV and DENV amplifications. Fifty microliters of virus stocks prepared were added to the flask and homogenized. The flask was incubated at 37 °C for 1 h. The volume of the flask was made to 25 mL with DMEM and 2% FBS supplemented with 1% Pen/Strep and incubated until the cytopathic effect was 90%. Infected cell supernatants were harvested in 15 mL Falcon tubes, centrifuged at 2500 rpm for 5 min, and stored at −80 °C. All procedures were carried out using sterile techniques and in a biosafety cabinet.

### 2.2. Patient Sera Samples

**Figure 1:**
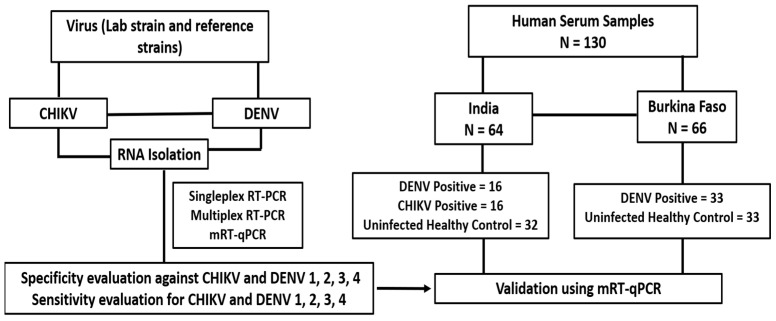
Study design and details of the human serum samples used in the study.

A total of 130 human serum samples were used in the present study after obtaining informed consent from the patients in both India and Burkina Faso. Among the 130 serum samples, 32 were positive for DENV (*n* = 16), and CHIKV (*n* = 16) and 32 from uninfected individuals (negative for both viruses) were part of a study funded by the government of India [29]. Also, 33 of the 130 serum samples were positive only for DENV, and 33 serum samples were negative for DENV, kindly offered by the National Institute of Public Health, Burkina Faso (Figure 1). Sera samples that were positive for the respective viruses by real-time RT PCR were selected for the study [29]. The protocol of this study was reviewed and approved by the ethical committees of the institutional ethical committees of the Health Science Research, Burkina Faso No. A026-2023/CEIRES/IRSS, and ICGEB, New Delhi, ICGEB/IEC/2014/01 version 3.

### 2.3. Primer Design

Nucleotide sequences for the complete genome of each serotype of the dengue virus were downloaded from the National Center for Biotechnology Information (NCBI) database and aligned to identify highly conserved regions using MEGA Software version 11. DENV 1–4 primers were designed manually; forward conserved primers were designed for all serotypes; and reverse primers of specific serotypes were designed using only specific conserved regions of different serotypes of DENV. A new DENV-3 forward (DENV-3 q F) and the reverse DENV-3 R have been designed by the multi-alignment of another genome of four DENV serotypes for RT-qPCR use. The representative sequences of CHIKV were downloaded from the Virus Pathogen Database and Analysis Resource (ViPR), and primers were designed to target the E1 gene using Snap Gene version 5.3.1. All RT-PCR primers were designed using similar parameters so that they would have similar melting temperatures (Tm). The potentials for dimerization and secondary structures were analyzed using Oligo Evaluator^TM^ online software . The length of all amplicons was 200 bp DENV-1, 367 bp DENV-2, 1359 bp DENV-3, 118 bp DENV-4, and 574 bp CHIKV. The specificity of all primer sequences was further confirmed using primer BLAST (NCBI). Detailed information on primers is provided in Table 1, and genome nucleotide accession numbers are provided in the Supplementary Materials.

### 2.4. Viral RNA Extraction

Viral RNA was extracted from a 150 µL cell culture supernatant and clinical samples using a NucleoSpin RNA Virus kit (MACHEREY-NAGEL Gmbh Co. KG., Düren, Germany), according to the manufacturer’s instructions, and eluted in 50 µL of diethyl pyrocarbonate (DEPC)-treated water. Purified RNA was quantified using Nanodrop 2000 and cryopreserved at −80 °C until further processing.

### 2.5. One-Step Multiplex RT-PCR Amplification

A multiplex RT-PCR assay was optimized for the simultaneous detection and serotyping of DENV and CHIKV RNAs in cell culture supernatants and human sera. mRT-PCR was performed using the PrimeScript One Step RT-PCR Kit (Takara Bio-INC, Shiga, Japan). A total volume of 15 µL of the reaction mixture consisting of 100 ng of extracted RNA, 7.5 µL of 2X RT buffer, 0.6 µL of the enzyme, 0.45 µL (10 µM) of the forward conserved primer (DENV-1, DENV-2, DENV-3, and DENV-4), 0.11 µL (10 µM) of the forward primer of CHIKV, 0.3 µL (10 µM) of the reverse primers of DENV-1 and DENV-4, and 0.11 µL (10 µM) of the reverse primers of DENV-2, DENV-3, and CHIKV; the reaction was complete to 15µL with DEPC water. DEPC water was used as a negative control. The thermal cycling profile of this assay consists of a 30 min reverse transcriptase (RT) step, which is performed at 50 °C, 95 °C for 5 min, and then 35 cycles of 95 °C for 30 s, 62 °C for 20 s, 72 °C for 30 s, and final extension 72 °C for 5 min. mRT-PCR was performed using an Applied Biosystems ProFlex™ 3 × 32-well PCR System machine. The PCR products were then analyzed by gel electrophoresis. A total of 15 µL of PCR product was loaded into a 1% (*W*/*V*) agarose gel in 1x Tris-Acetate-EDTA buffer with a 1 Kbp ladder as a molecular weight marker. To detect coinfections between DENV 1–4 and CHIKV, individual serotype RNAs of the reference sample were mixed in equal quantities, and 3 µL was used for mRT-PCR detection.

### 2.6. Multiplex One-Step Real-Time RT-PCR

A real-time, one-step, multiplex SYBR Green I RT-PCR assay was also developed for the detection of DENV-1, DENV-4, and CHIKV. This assay was performed on a PikoReal 96 Real-Time PCR System machine (Thermo Scientific, Waltham, MA, USA) using a one-step QuantiTect SYBR Green kit (Qiagen, Hilden, Germany). All mRT-qPCR reactions were performed in 50 µL reactions with 25 µL of SYBR Green, 0.5 µL of the enzyme, 1.4 µL (10 µM) of the forward conserved primer (DENV-1, DENV-2, and DENV-4), 0.46 µL (10 µM) of the DENV-3 q forward and reverse primers, 0.47 µL (10 µM) of the CHIKV forward and reverse primers, 0.5 µL (10 µM) of the reverse primer of DENV-1 and DENV-4, 0.44 µL (10 µM) of the DENV-2 reverse primer, and 100 ng of RNA template. A total of 15 µL of reaction was used in triplicate for all viruses. DEPC water was used as a negative control. The RT-PCR conditions for real-time RT-PCR consisted of a 30-min RT step at 50 °C and 10 min of Taq polymerase activation at 95 °C, followed by 40 cycles of PCR at 95 °C for 30 s (denaturation), 62 °C for 30 s (annealing), 72 °C for 30 s (extension), and final extension at 60 °C for 30 s. The melting curve temperature ranged from 60 °C to 95 °C. The result was positive if the cycle threshold (C_t_) values were equal to or less than 33 cycles. If C_t_ is more than 33 cycles, the result is considered negative. The limit of sensitivity of the assay was carried out with known quantitative RNA standards prepared using the one-step QuantiTect SYBR Green method. Briefly, the RNA template of each virus was serially diluted 10-fold or 100-fold with the known concentrations of DENV 1–4 and CHIKV, and 4 µL (for ten-fold) or 5 µL (for 100-fold) of the diluted RNA were added to the mRT-qPCR reaction tube and amplified in triplicate. Subsequently, standard curves were drawn, and LOD was determined using these standard curves. Melting curve analysis was performed after PCR amplification to verify that the correct product was amplified by examining its specific melting temperature (Tm), which was also used to serotype DENV.

After optimization, the positive RNA of DENV and CHIKV and the negative RNA from pre-collected sera were assayed in the same condition to evaluate the multiplex RT-PCR and RT-qPCR for their diagnostic potential in a clinical sample and for validation. In Burkina, the clinical sample was performed using an Applied Biosystems (SimpliAmp Thermo Fisher Scientific, Waltham, MA, USA) thermal cycler for mRT-PCR and the CFX96 Real-Time System (BIO-RAD, Hercules, CA, USA) for mRT-qPCR.

### 2.7. Sequence Analysis

After gel electrophoresis, the amplicon of the different viruses was cut and purified using the NucleoSpin^®^ Gel and PCR Clean-up Kit (MACHEREY-NAGEL Gmbh Co. KG., Düren, Germany) according to the manufacturer’s instructions and eluted in 25 µL NE buffer. Twenty microliters of purified PCR product were sent to Macrogen (Seoul, Republic of Korea) for deoxyribonucleic acid (DNA) sequencing by the Sanger method using the amplification primers. Forward and reverse sequences were assembled by BioEdit software version 7.2.5.0, to determine consensus sequence and blast using the NCBI BLAST tool to confirm the specificity and accuracy of the amplification of different viral RNA fragments.

## 3. Results and Discussion

### 3.1. Primer Design and Their Specificity Assessment for Multiplex Detection of DENV 1–4 and CHIKV

Clinical manifestations of disease caused by CHIKV and DENV are indistinguishable in their early phases and very difficult to differentiate by serological testing. To bridge the caveat, we opted to design multiplex viral RNA based assays using RT-PCR and RT-qPCR. For the development of these multiplex RT-PCR and RT-qPCR assays for the simultaneous detection of all DENV serotypes and CHIKV as co-infection, specific attributes for primer design were employed, making use of geographically specific virus sequences. Sets of primers were designed based on the optimal conserved regions revealed by multiple sequence alignments of three complete genomes of all DENV serotypes (DENV-1 to DENV-4) using MEGA software (Supplementary Figure S1). A universal forward primer was designed from a conserved region of the DENV-1 to DENV-4 genome to ensure the detection of all four DENV serotypes; the reverse primers of all serotypes were designed using only specific conserved regions of different serotypes of DENV, but for improving mRT-qPCR detection, another DENV-3 forward primer (DENV-3 q F) was designed to be used in SYBR Green real-time PCR (Supplementary Figure S1). CHIKV primers were designed using SnapGene by targeting the E1 gene. Compatible melting temperatures (average Tm ranging from 60 to 65 °C) were selected from the primer set.

**Figure 2:**
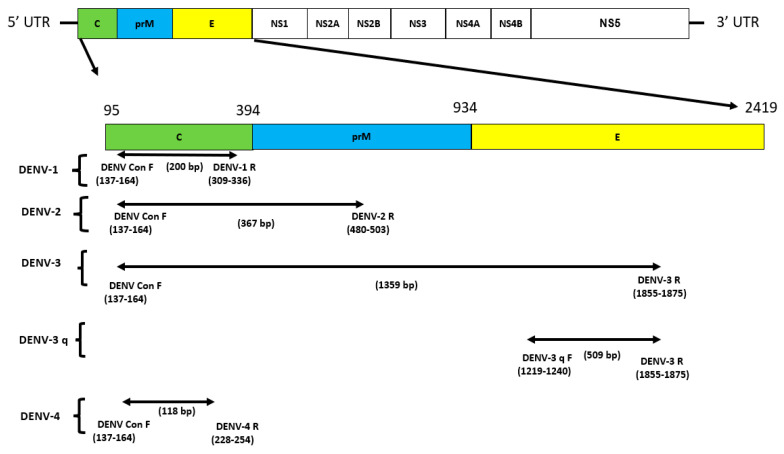
The DENV genome and primers targeted regions used in this multiplex detection study. Structural proteins are targeted in this study for DENV: DENV conserve forward is located in the capsid (C) protein, reverse DENV–1 and DENV–4 in the C protein, and reverse DENV–2 in the precursor membrane prM protein. DENV–3 q forward and reverse DENV–3 in envelope protein E; UTR: untranslated region.

Of the different conserved regions selected for primer design, DENV conserved forward was located in the capsid (C) protein, reverse DENV-1 and DENV-4 in the C protein, reverse DENV-2 in the precursor membrane prM protein, and DENV-3 q forward and reverse DENV-3 in the envelope E protein (Figure 2). The oligonucleotide sequences and genome positions of all primers for the multiplex assay are listed in Table 1.

**Figure 3:**
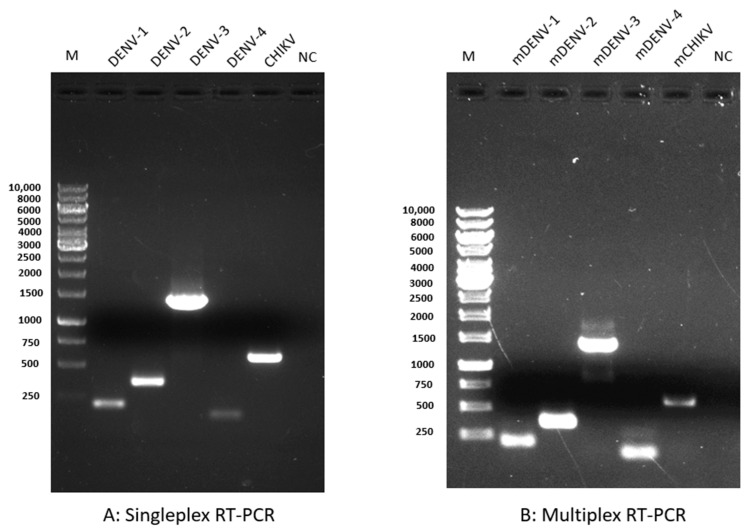
Comparison of the sensitivity and specificity of singleplex and multiplex RT-PCR. Electrophoresis of a 1% agarose gel loaded with 15 µL of singleplex RT-PCR and multiplex RT-PCR products shows the specificity of the primers. (**A**) Singleplex RT-PCR: lane 1, M (1 kb DNA marker); lane 2, DENV-1 (200 bp); lane 3, DENV-2 (367 bp); lane 4, DEN-3 (1359 bp); lane 5, DENV-4 (118); lane 6, CHIKV (574 bp); and lane 7, negative control (NC). (**B**) Multiplex RT-PCR: lane 1, M (1 kb DNA marker); lane 2, mDENV-1 (200 bp); lane 3, mDENV-2 (367 bp); lane 4, mDEN-3 (1359 bp); lane 5, mDENV-4 (118); lane 6, mCHIKV (574 bp); and lane 7, NC. m: multiplex; bp: basis pair; and NC: negative control.

DENV 1–4 and CHIKV-specific regions were amplified successfully by multiplex RT-PCR and RT-qPCR assays, and their amplicons were further observed on agarose gel electrophoresis for confirmation based on the amplicon size and real-time detection based on the cycle threshold (C_t_) value. C_t_ were DENV-1 (C_t_ = 17.28), DENV-2 (C_t_ = 23.5), DENV-3 (C_t_ = 22.06), DENV-4 (C_t_ = 21.73), and CHIKV (C_t_ = 22.75) (Table 2 and Figure 3). The expected size of the amplicons was 200 bp DENV-1, 367 bp DENV-2, 1359 bp DENV-3 (RT-PCR only), 118 bp DENV-4, and 574 bp CHIKV.

The amplicon size of DENV-3 used in real-time RT-PCR was 509 bp. We observed a single specific band corresponding to the selected virus in the multiplex RT-PCR. We did not observe any cross-reactivity of CHIKV-specific primers with any DENV or vice versa. Moreover, we also included the genomic DNA of *Plasmodium falciparum* and the RNA of SARS-CoV-2 in our multiplex RT-PCR and did not observe cross-reactivity.

**Figure 4:**
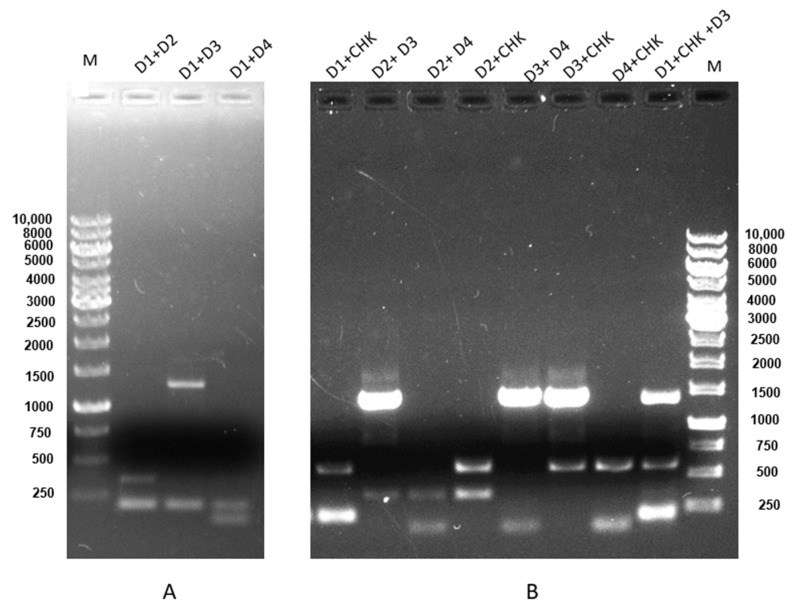
Coinfection and concurrent infection detection between DENV 1–4 and CHIKV. (**A**) Concurrent infection using an RNA mixture of DENV-1 (200 bp) + DENV-2 (367 bp), lane 2; DENV-1 (200 bp) + DENV-3 (1359 bp), lane 3; DENV-1 (200 bp) + DENV-4 (118), lane 4; M is 1 kb DNA marker. (**B**) Coinfection using an RNA mixture of DENV-1 (200 bp) + CHIKV (574 bp), lane 1; DENV-2 (367 bp) + DENV-3 (1359 bp), lane 2; DENV-2 (367 bp) + DENV-4 (118), lane 3; DENV-2 (367 bp) + CHIKV (574 bp), lane 4; DENV-3 (1359 bp) +DENV-4 (118), lane 5; DENV-3 (1359 bp) + CHIKV (574 bp), lane 6; DENV-4 (118) + CHIKV (574 bp), lane 7; and DENV-1 (200 bp) + DENV-3 (1359 bp) + CHIKV (574 bp), lane 8; M is a 1 kb DNA marker. Electrophoresis of a 1% agarose gel loaded with a 15 µL product showed the specificity of the primers in coinfection detection with mRT-PCR.

Furthermore, the sequence of all viruses was confirmed by subsequent DNA sequencing results and BLAST analysis (Supplementary Figure S2), confirming the sensitivity and specificity of the assay. Evaluation of coinfection of all possible combinations amongst DENV 1–4 and CHIKV indicates that our mRT-PCR assay could simultaneously detect and serotype DENV and CHIKV in a single reaction (Figure 4).

Several studies have previously reported distinct molecular assays developed using TaqMan real-time RT-PCR techniques or SYBR green base with universal DENV primers and RT-PCR with other reverse transcriptase for detecting dengue serotypes as well as other flaviviruses [11,24,25]. Similarly, commercial rapid diagnostic tests (RDTs) such as SD BIOLINE Chikungunya IgM, RTK ProDetect^TM^, and SD BIOLINE Dengue Duo^®^ are available for detecting both CHIKV and DENV serotypes [30,31]. However, reports of false-positive results have dampened the enthusiasm for the use of these kits by clinicians. These reports also emphasize the need to design robust primer sets for efficient and stringent detection of viruses, a feature employed in the current study. For instance, primer design was performed according to several parameters in order to avoid heterodimer formation likelihood and thermal compatibility and to increase the sensitivity and specificity of our assay. Further, DENV serotyping primers were designed based on the genome alignment of multiple strains of all four serotypes of DENV both globally as well as taking country specific sequences to study the variations at the genes critically. The alignment of several sequences assured the improvement of the universal primers, mainly by the elimination of potential mismatches, increasing the possibility of amplification of templates with greater sequence diversity. Information on genes, such as C, prM, and E domains, used in earlier similar studies was considered for designing the primers [11,32,33].

### 3.2. Multiplex RT-qPCR Assessment and Sensitivity

mRT-qPCR was performed with 100 ng of RNA, and to evaluate sensitivity, 10-fold and 100-fold serial dilutions of RNA standards were used to draw a standard curve and estimate the lower limits of detection (LOD) of viral RNA copy number for the developed one-step real-time RT-PCR assay. C_t_ values obtained for serial 10-fold dilutions of known concentrations of DENV 1–4 and CHIKV RNA have been used to draw a linear curve against the amounts of standard RNA copy numbers and were used to calculate the correlation coefficient (Supplementary Figure S3). The details are provided in Table 2.

The limits of detection were calculated to be 4.1 × 10^−1^ copies/reaction for DENV-1, DENV-3, and CHIKV and 4.1 × 10^1^ for DENV-2 and DENV-4 (Table 2). The results showed that the multiplex one-step real-time RT-PCR assays could detect simultaneous DENV 1–4 and CHIKV in one reaction. 

The efficiency of the assays is supported by appropriate R^2^ (R^2^ = 0.99 for DENV-2 and DENV-3, R^2^ = 0.98 for DENV-4 and CHIKV, and R^2^ = 0.93 for DENV-1); our results are similar to previously published studies [25,34].

### 3.3. Performance of mRT-PCR and mRT-qPCR Assays on Clinical Samples and Validation

To determine the performance of multiplex RT-PCR and real-time RT-PCR assays, 32 human sera samples from patients infected with DENV and CHIKV and 32 human sera samples from individuals negative for these viruses from India were used. Also, 33 clinical samples positive for DENV and 33 samples from uninfected serum pre-collected at the National Institute of Public Health, Burkina Faso, were used. All clinical samples were already tested for their infection status through RT-PCR and serology and were used for multiplex detection with mRT-PCR and mRT-qPCR. Among India clinical samples, the mRT-PCR assay was able to detect 87.5% (14/16) samples for DENV and 93.7% (15/16) for CHIKV, whereas the mRT-qPCR assay was able to detect 100% (16/16) samples for DENV and 100% (16/16) for CHIKV; serotyping showed that 43.7% (7/16) samples were positive for DENV-1, 12.5% (2/16) were positive for DENV-2, 37.5% (6/16) were positive for DENV-3, and 6.3% (1/16) were positive for DENV-4. In Burkina, clinical sample positives for DENV were 90.9% (30/33) were positives by mRT-PCR and 100% (33/33) by mRT-qPCR (Table 3); according to serotyping, 57.5% (19/33) samples were DENV-1 and 42.5 (14/33) for DENV-3. The sensitivity of mRT-PCR and mRT-qPCR is 91% and 100%, respectively. All positive samples tested by mRT-PCR were also positive for real-time RT-PCR. The mRT-qPCR assay was seen to be more sensitive than the conventional multiplex RT-PCR. The results and the comparison of detection rates between both assays are summarized in Table 1. We have used clinical sample negatives for DENV and CHIKV to evaluate the specificity of both conventional multiplex RT-PCR and SYBR Green real-time RT-PCR assays. A 100% specificity was found for both assays (Table 3).

Two multiplex assays were validated using positive clinical samples pre-collected in India and Burkina Faso. The sensitivity and specificity of the conventional multiplex RT-PCR assays are 91% and 100%, respectively, and for the real-time SYBR Green assay, 100% and 100%, respectively. mRT-qPCR is more sensitive than mRT-PCR. In the study by Chen et al., an evaluation of the SYBR Green-I-based one-step multiplex real-time RT-PCR assay was performed according to the DENV serotype; the sensitivity for DENV-1, DENV-2, DENV-3, DENV-4, and CHIKV was 89.66%, 96.67%, 96.67%, 94.12%, and 95.74%, respectively, with 100% specificity [25]. In our study, evaluations were performed according to the samples positive for DENV. The sensitivity of mRT-PCR and the real-time SYBR Green assay is similar to the study of Chen et al. Likewise, another report [11] (11) previously developed a multiplex RT-PCR (mRT-PCR) assay for simultaneous detection and serotyping of DENV and CHIKV [11]. However, in their study, a combination of DNA polymerase and reverse transcriptase was used to achieve comparable sensitivity, thereby increasing the total cost per sample.

In our study, we developed two molecular assays using a single enzyme and a single step reaction in a single tube, making it cost-effective for the rapid detection of DENV serotypes and CHIKV. The combination of these tools offers high sensitivity and specificity. These results show that mRT-PCR and mRT-qPCR can be used in Burkina Faso for DENV and CHIKV surveillance. Economic evaluation has shown that our molecular tool detection cost per sample is around USD 6 for mRT-PCR and around USD 8 for mRT-qPCR, whereas the TaqMan real-time RT-PCR technique detection cost is around USD 20. Our molecular tools are low-cost compared to TaqMan real-time RT-PCR techniques. In Burkina Faso, DENV is the main endemic arbovirus included in the diagnostic and surveillance protocols, which explains the difficulty of obtaining CHIKV-positive samples. Only one seroprevalence study on CHIKV has been documented [16]. These multiplexed methods will help to improve early detection and effective surveillance of CHIKV in both humans and vectors.

## 4. Conclusions

Differential diagnosis is key to effective disease and patient management. Dengue serotyping gives evidence of the displacement of the dominant circulating serotypes across time and plays an important role in predicting the severity of future outbreaks [35]. From an international perspective, accurate serotyping will allow a better understanding of traveling waves in dengue fever transmission by identifying related outbreaks across borders [36]. In addition, serotyping data can inform research into the multi-annual cross-country periodicity of dengue, thought to be related to the cycling of host immunity to different serotypes [37]. The present study is a proof of the concept of successfully employing multiplex PCR for efficient detection of CHIKV and DENV serotypes using SYBR Green PCR chemistry and serves as a cost-effective option for use in resource-constrained countries in Africa and Asia, both for the diagnosis and epidemiologic surveillance of CHIKV and DENV.

## Figures and Tables

**Table 1:** Oligonucleotide primers used in multiplex RT-PCR and RT-qPCR assays.

Family/Genus	Virus	Primer	Sequence (5′ to 3′)	Target	Genome Position
*Flaviviridae*/*Flavivirus*	DENV	DENVcon F	TCAATATGCTGAAACGCGAGAGAAACCG	C	137–164
		DENV-3 q F	AGGAGCTACGTGGGTTGACGTG	E	1219–1240
		DENV-1 R	TTTGATCGCTCCATTCTTCTTGAATGAG	C	309–336
		DENV-2 R	TTCCCTTTCTCTTGTCTACTGACG	prM	480–503
		DENV-3 R	GTGGTGAGCATTCTAGCCCAA	E	1855–1875
		DENV-4 R	GTGATGAATGCTAGCACCATCCGTAAG	C	228–254
*Togaviridae*/*Alphavirus*	CHIKV	CHIKV F	ACACGTAAACAGTGATCCCGAACAC	E1	9998–10,022
		CHIKV R	CCAAACGGCGGGTAGTCCATGT		10,550–10,571

DENVconF: dengue virus conserved forward; R: reverse; DENV-3 q F: DENV-3 forward for RT-qPCR using GenBank access: DENV-1 (NC_001477.1, MN869914.1, MT929577.1, KC692515.1, KC692512.1, and JN697057.1); DENV-2 (AY858035.2, AY858036.2, AB189124.1, FM210237.2, FM210232.2, and FM210227.1); DENV-3 (AB189127.1, AY858044.2, AY858048.2, KF954948.1, ON123669.1, and KC762688.1); DENV-4 (KF955510.1, HQ332172.1, HQ332176.1, JX024758, OK605599.1, and KC762698.1); CHIKV (NC_004162.2).

**Table 2:** Sensitivity of DENV 1–4 and CHIKV in mRT-qPCR.

Parameter	mRT-qPCR				
	DENV-1	DENV-2	DENV-3	DENV-4	CHIKV
C_t_ value	17.28	23.5	22.06	21.73	22.75
R^2^	0.93	0.99	0.99	0.98	0.98
LOD (copies/reaction)	4.1 × 10^−1^	4.1 × 10^1^	4.1 × 10^−1^	4.1 × 10^1^	4.1 × 10^−1^
Tm	79.17	80.01	82.7	78.71	81.84

Tm: melting temperature; C_t:_ cycle threshold; R^2^: coefficient of correlation; and LOD: limit of detection.

**Table 3:** Diagnostic performance of mRT-qPCR and mRT-PCR in clinical samples.

Country	Sample	Total	mRT-qPCR + N (%)	mRT-qPCR − N (%)	mRT-PCR + N (%)	mRT-PCR − N (%)
**India**	DENV positive	16	16 (100.0)	0 (0.0)	14 (87.5)	2 (12.5)
	CHIKV positive	16	16 (100.0)	0 (0.0)	15 (93.7)	1 (6.3)
	Healthy Control (CHIKV and DENV negative)	32	0 (0.0)	32 (100.0)	0 (0.0)	32 (100.0)
**Burkina Faso**	DENV positive	33	33 (100.0)	0 (0.0)	30 (90.9)	3 (9.1)
	DENV negative	33	0 (0.0)	33 (100.0)	0 (0.0)	33 (100.0)
**Sensitivity**			100%		91%	
**Specificity**			100%		100%	

## Data Availability

Data are contained within the article and Supplementary Materials.

## Supplementary Materials

The following supporting information can be downloaded at: https://www.mdpi.com/article/10.3390/cimb46030134/s1, Figure S1. Multi-sequence alignment by MEGA and conserved region selection for DENV 1–4 primer design. The color sequence corresponds to the consensus-conserved region. (A): Conserved region selected for DENVcon F in position 137–164, DENV-1 R in position 309–336, DENV-2 R in position 480–503, and DENV-4 R in position 228–254; (B): Conserved region selected for DENV-3 q F is from position 1219–1240 and DENV-3 R to position 1855–1875 for mRT-qPCR Figure S2. Consensus sequence obtained by DNA sequencing and used for BLAST analysis of DENV 1–4 and CHIKV. (A) DENV-1 consensus sequence; (B) DENV-3 consensus sequence; (C) DENV-4 consensus sequence; (D) DENV-2 consensus sequence; (E) CHIKV consensus sequence. Forward and reverse sequences obtained by the Sanger sequencing method were assembled by BioEdit software, and consensus sequence BLAST in NCBI confirmed the specificity of all viruses detected in mRT-PCR; Figure S3. Standard curves of dengue virus DENV 1–4 and CHIKV in mRT-qPCR. Cycle threshold (C_t_) values obtained for serial 10-fold dilutions of known concentrations of DENV 1–4 and CHIKV RNA have been used to draw a linear curve against the amounts of standard RNA copy numbers (from 10^2^ to 10^0^ copies/µL). The assays were performed in triplicate; Figure S4. Standard curves of dengue virus DENV 1–4 and CHIKV in mRT-qPCR. Cycle threshold (C_t_) values obtained for serial 100-fold dilutions of known concentrations of DENV 1–4 and CHIKV RNA have been used to draw a linear curve against the amounts of standard RNA copy numbers. The assays were performed in triplicate. The dotted line represents the detection limit, i.e., the C_t_ value of 34.
