# Supplementary material for: Development of Multiplex Molecular Assays for Simultaneous Detection of Dengue Serotypes and Chikungunya Virus for Arbovirus Surveillance
Source: Curr Issues Mol Biol. 2024 Mar 6;46(3):2093–104. doi: 10.3390/cimb46030134 (PMC10969301; doi:10.3390/cimb46030134)
Supplement: Supplementary file 1 [file cimb-46-00134-s001.zip › cimb-2799665-supplementary.pdf]

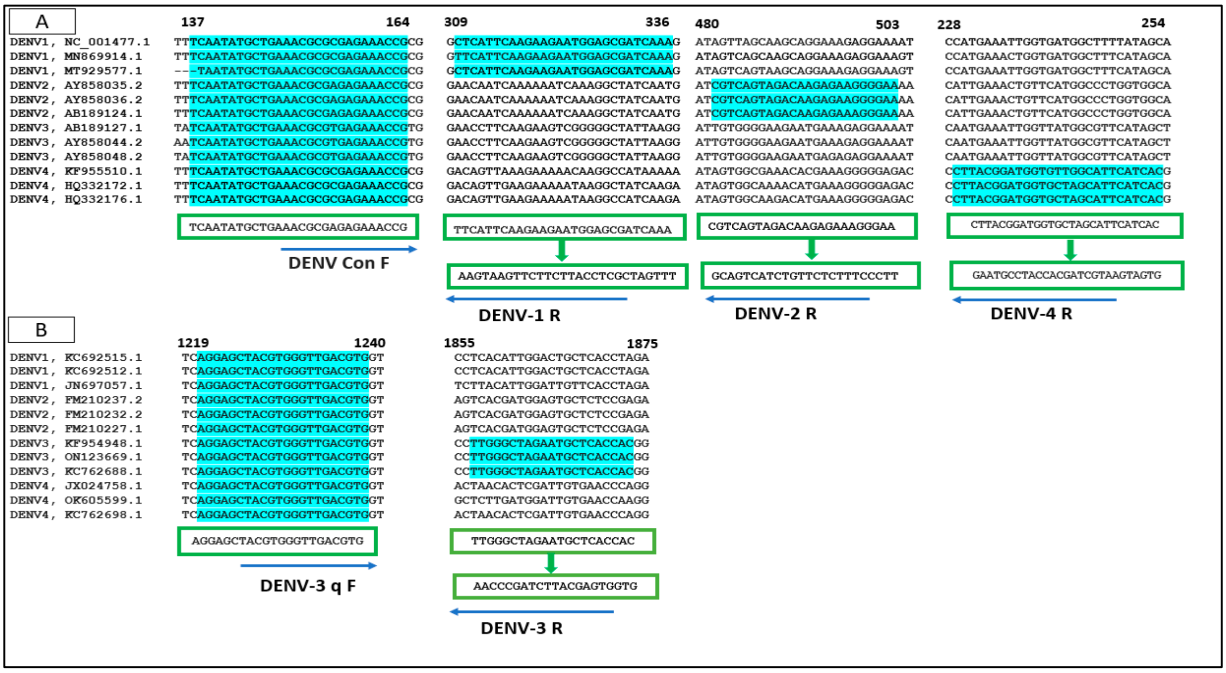

Figure S1. *Multi-sequence alignment by MEGA and conserved region selection for DENV1-4 primers design. The color sequence corresponds to the consensus conserved region. (A): Conserve region selected for DENVcon F in position 137-164, DENV-1 R in position 309-336, DENV-2 R in position 480-503, DENV-4 R in position 228-254; (B): Conserve region selected for DENV-3 q F in position 1219-1240 and DENV-3 R in position 1855-1875 for mRT-qPCR using.*

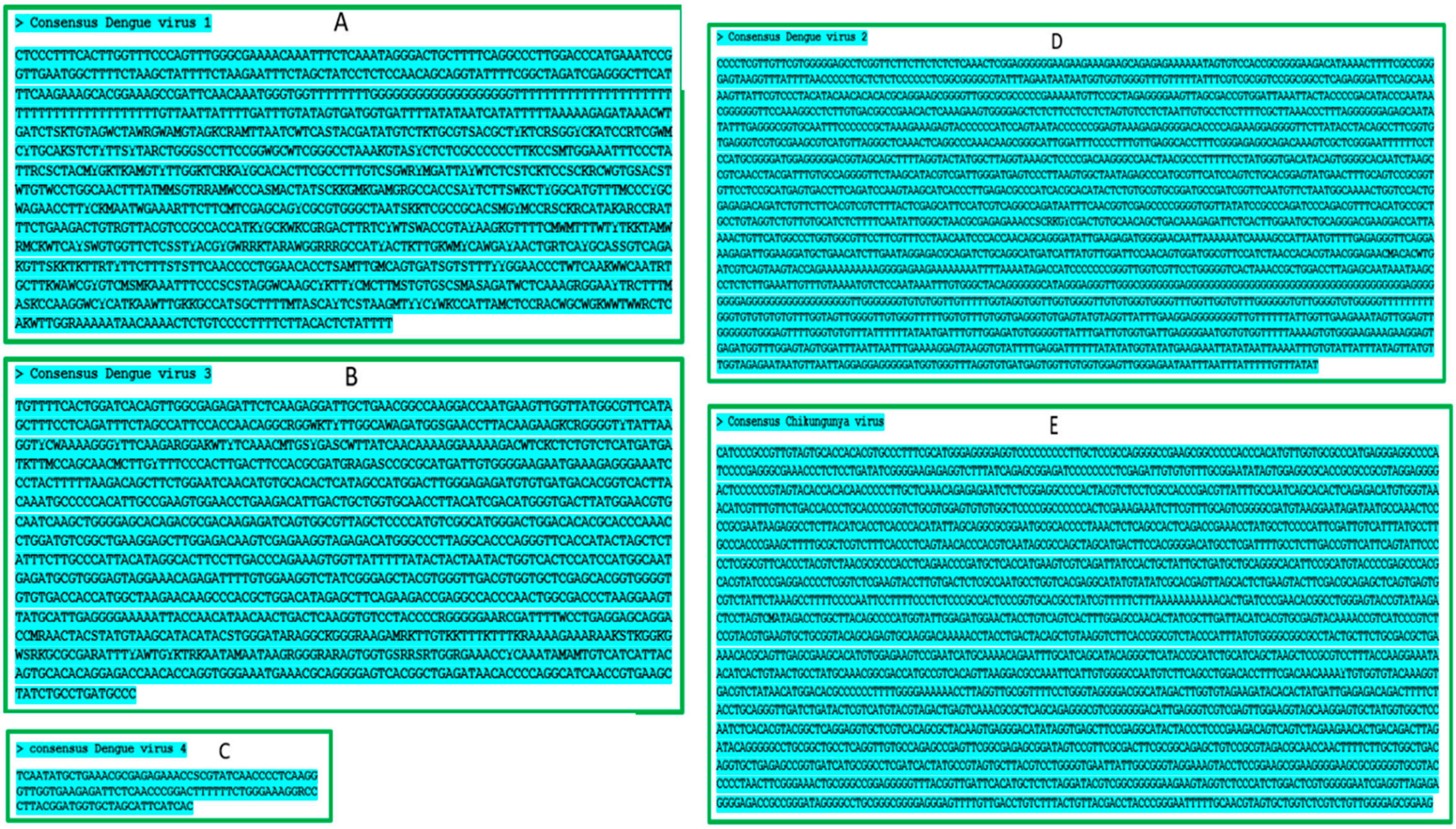

Figure S2*. Consensus sequence obtained by DNA sequencing and used for BLAST analysis of DENV1-4 and CHIKV.* ***(A)*** *DENV-1 consensus sequence;* ***(B)*** *DENV-3 consensus sequence;* ***(C)*** *DENV-4 consensus sequence;* ***(D)*** *DENV-2 consensus sequence;* ***(E)*** *CHIKV consensus sequence. Forward and Reverse sequences obtained by the Sanger sequencing method were assembled by BioEdit software and consensus sequence BLAST in NCBI confirmed the specificity of all viruses detected in mRT-PCR.*

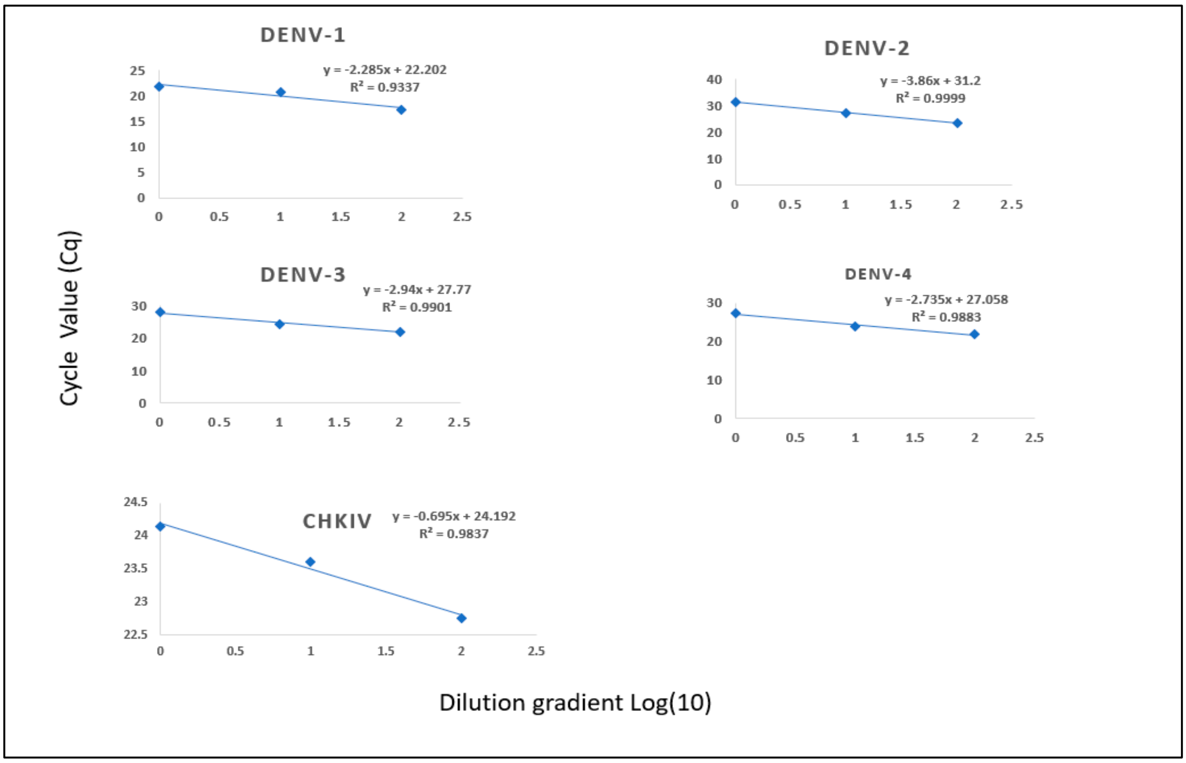

Figure S3. *Standard curves of dengue virus DENV 1-4 and CHIKV in mRT-qPCR. Cycle threshold (C_t) values obtained for serial 10-fold dilutions of known concentration of DENV 1-4 and CHIKV RNA has been used to draw a linear curve against the amounts of standard RNA copy number (from 10^2 to 10^0 copies/µL). the assays were performed in triplicate.*

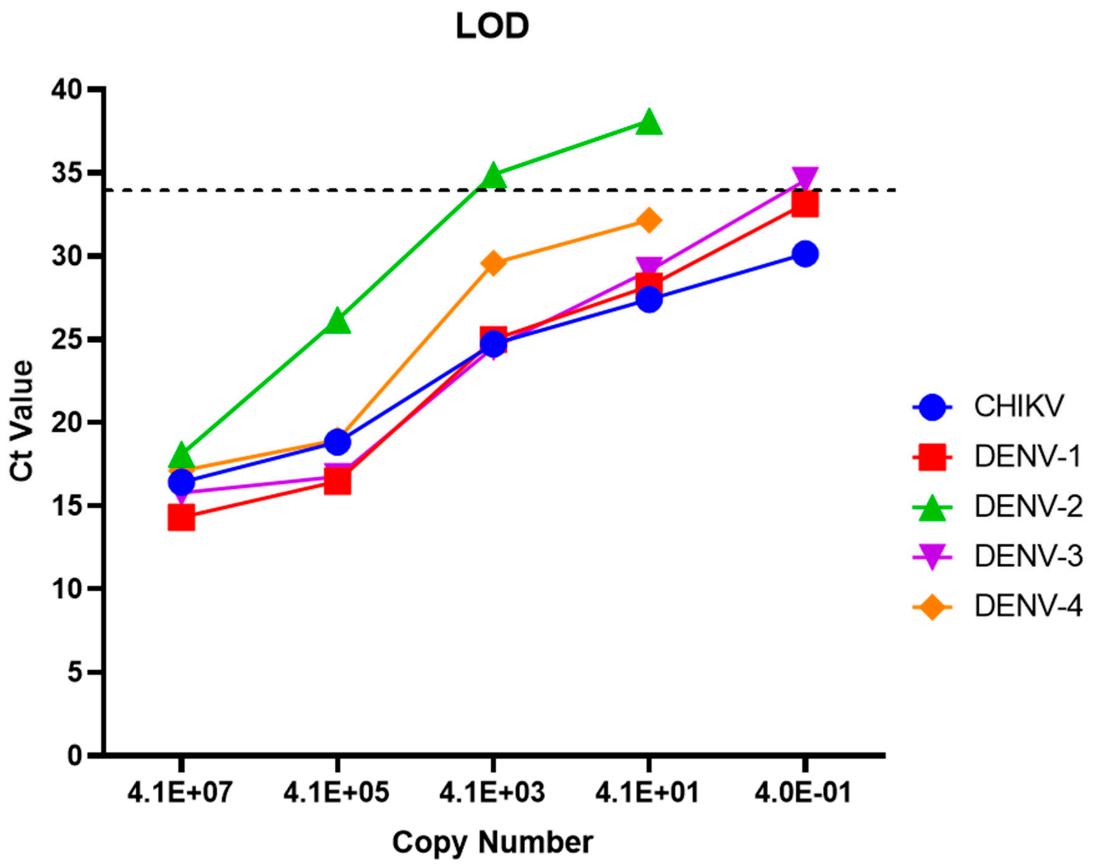

Figure S4: *Standard curves of dengue virus DENV 1-4 and CHIKV in mRT-qPCR. Cycle threshold (C_t) values obtained for serial 100-fold dilutions of known concentration of DENV 1-4 and CHIKV RNA has been used to draw a linear curve against the amounts of standard RNA copy number. the assays were performed in triplicate. The dotted line represents the detection limit i.e. Ct value of 34.*
